# Active pH regulation facilitates *Bacillus subtilis* biofilm development in a minimally buffered environment

**DOI:** 10.1128/mbio.03387-23

**Published:** 2024-02-13

**Authors:** Peter Tran, Stephen M. Lander, Arthur Prindle

**Affiliations:** 1 Department of Chemical and Biological Engineering, Northwestern University, Evanston, Illinois, USA; 2 Center for Synthetic Biology, Northwestern University, Evanston, Illinois, USA; 3 Medical Scientist Training Program, Feinberg School of Medicine, Northwestern University, Chicago, Illinois, USA; 4 Department of Biochemistry and Molecular Genetics, Feinberg School of Medicine, Chicago, Illinois, USA; Universite de Geneve, Geneva, Switzerland

**Keywords:** biofilms, microbial communities, pH, buffer, emergent behaviors

## Abstract

Biofilms provide individual bacteria with many advantages, yet dense cellular proliferation can also create intrinsic metabolic challenges including excessive acidification. Because such pH stress can be masked in buffered laboratory media—such as MSgg commonly used to study *Bacillus subtilis* biofilms—it is not always clear how such biofilms cope with minimally buffered natural environments. Here, we report how *B. subtilis* biofilms overcome this intrinsic metabolic challenge through an active pH regulation mechanism. Specifically, we find that these biofilms can modulate their extracellular pH to the preferred neutrophile range, even when starting from acidic and alkaline initial conditions, while planktonic cells cannot. We associate this behavior with dynamic interplay between acetate and acetoin biosynthesis and show that this mechanism is required to buffer against biofilm acidification. Furthermore, we find that buffering-deficient biofilms exhibit dysregulated biofilm development when grown in minimally buffered conditions. Our findings reveal an active pH regulation mechanism in *B. subtilis* biofilms that could lead to new targets to control unwanted biofilm growth.

**IMPORTANCE:**

pH is known to influence microbial growth and community dynamics in multiple bacterial species and environmental contexts. Furthermore, in many bacterial species, rapid cellular proliferation demands the use of overflow metabolism, which can often result in excessive acidification. However, in the case of bacterial communities known as biofilms, these acidification challenges can be masked when buffered laboratory media are employed to stabilize the pH environment for optimal growth. Our study reveals that *B. subtilis* biofilms use an active pH regulation mechanism to mitigate both growth-associated acidification and external pH challenges. This discovery provides new opportunities for understanding microbial communities and could lead to new methods for controlling biofilm growth outside of buffered laboratory conditions.

## INTRODUCTION

Bacteria inhabit a diverse range of environmental niches and engage in specific lifestyles to thrive within their local environment. In controlled laboratory conditions, bacteria primarily exist as planktonic (free-swimming) individuals, whereas in natural environments, bacteria often form sessile, multicellular communities known as biofilms (1, 2). Biofilms create a densely packed local environment with extracellular matrix (ECM) (3–5) that can give rise to complex emergent behaviors such as cell-to-cell signaling (6, 7), macroscopic spatiotemporal organization (8–10), and metabolic remodeling (11, 12). Furthermore, the biofilm structure creates a diffusion barrier and resulting local concentration gradients, producing habitat diversity and increased resilience against antibiotics (13–15). Thus, the biofilm state confers advantages to individual bacteria for persisting in their local environment that are unavailable to planktonic cells.

However, this dense cellular proliferation can also create unique metabolic challenges. In particular, rapidly growing biofilm bacteria engage in overflow metabolism where carbon is not completely oxidized via respiration and instead only partially oxidized via fermentation (16, 17). This counterintuitive strategy enables rapid growth by circumventing production of energy-intensive respiratory enzymes while using increased metabolic flux into fermentation pathways that produce excretable byproducts such as lactate and acetate (16, 18). In the densely packed and diffusion-limited biofilm environment, these acidic metabolites can accumulate and exacerbate metabolic stress on sessile biofilm cells (19, 20). Importantly, excessively acidic conditions disrupt a cell’s ability to maintain functional proton motive force (PMF), increase energy expenditure for maintaining intracellular pH homeostasis, and impede growth via degradation of enzymatic activity (21–23). These effects may be particularly pronounced for Gram-positive bacteria that possess only a single cell membrane where the electron transport chain (ETC) is directly exposed to extracellular pH (24). Consequently, biofilm cells must maintain pH homeostasis against increasingly acidic conditions that arise during biofilm development.

These acidification challenges can be masked when buffered laboratory media are employed to stabilize the pH environment for optimal bacterial growth (25, 26). In contrast, bacteria in nature persist in settings that often lack robust buffering systems and face significant pH variation from environmental sources and heterogeneous mixing (1, 2, 27). Indeed, natural environments, such as the soil, ocean, and human gastrointestinal tract, exhibit pH gradients that can influence microbial population composition and behaviors (28–31). It, therefore, remains unclear how biofilms maintain growth, PMF, and pH homeostasis against the acidification associated with biofilm growth in such minimally buffered environments. To approach this question, we established a biofilm model system with a minimally buffered media that preserves cellular growth while enabling measurement of the local pH. Our findings reveal that *B. subtilis* biofilms use an active pH regulation mechanism to both facilitate biofilm development and tolerate acidification in minimally buffered conditions.

## RESULTS

**Fig 1:**
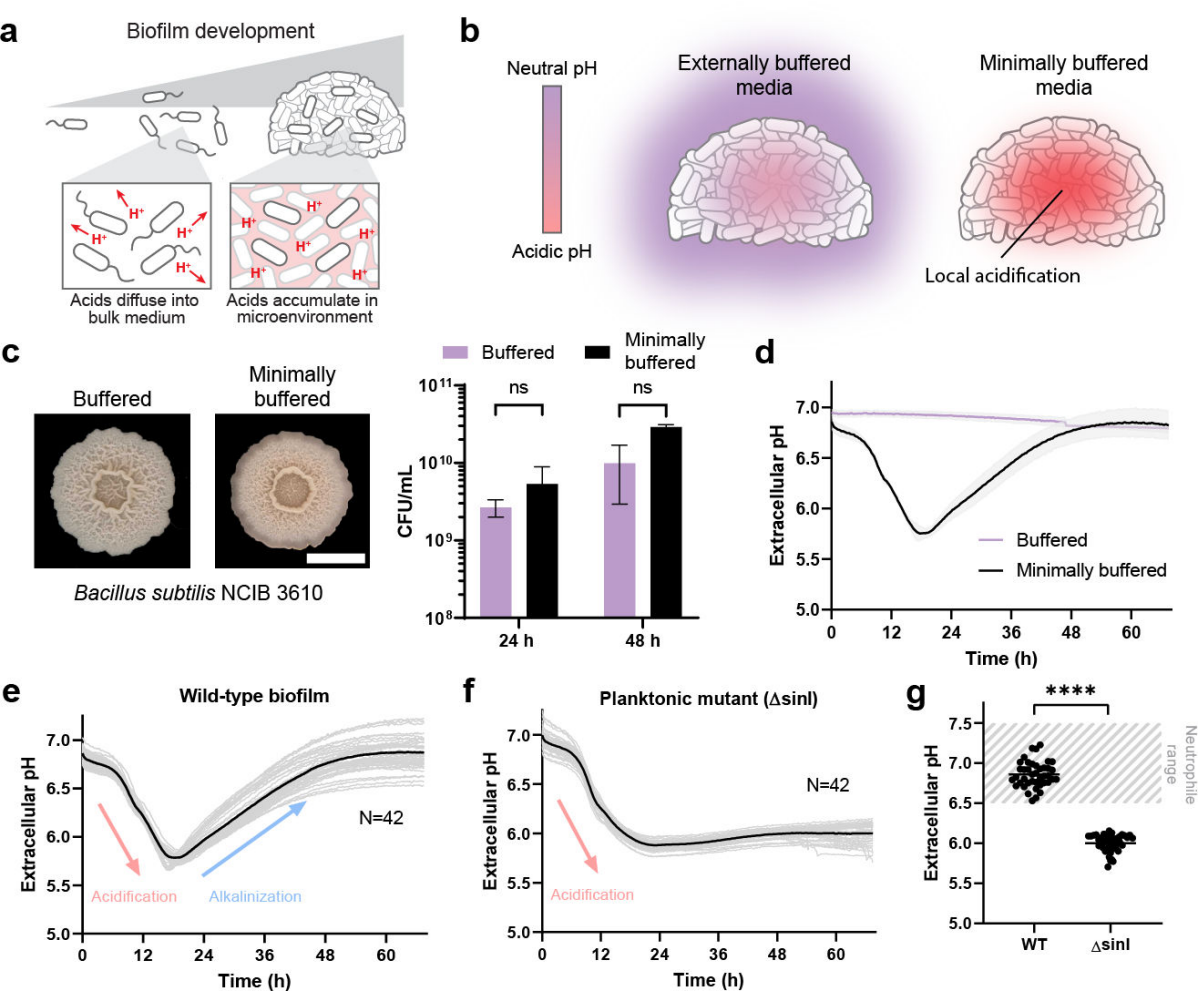
Modulation of extracellular pH by *B. subtilis* biofilms in minimally buffered conditions. (**a)** Schematic showing biofilm development and acidification of the local environment.** (b)** Schematic showing measurements of extracellular pH in biofilms across different buffered environments using a cell-impermeable pH reporter, 2′,7′-bis-(carboxyethyl)-5-(and-6)-carboxyfluorescein (BCECF) free acid. (c) Left, images of *B. subtilis* NCIB 3610 grown on fully buffered MSgg media containing 100 mM MOPS vs minimally buffered. Images correspond to biofilms at 48 h; scale bar represents 5 mm. Right, colony-forming unit (CFU) measurements of 3610 biofilms grown on buffered and minimally buffered MSgg. Biofilms were harvested at 24 and 48 h. Images correspond to biofilms at 48 h; scale bar represents 5 mm. Data: mean ± SD, *n* = 3 technical replicates. Strain: NCIB 3610. (**d)** Extracellular pH tracked over time with NCIB 3610 biofilms in buffered (100 mM MOPS) and minimally buffered (1 mM MOPS) MSgg. Biofilms were grown statically at 30°C. Extracellular pH is calculated from the fluorescence intensity (535 nm) of a cell-impermeable pH reporter, BCECF free acid, over time. Data: mean ± SD, *n* = 4 technical replicates. Strain: NCIB 3610.** (e)** Extracellular pH measurements for NCIB 3610 wild-type (WT) biofilms. Data: mean ± SD, *n* = 42 technical replicates. Strain: NCIB 3610. (**f)** Extracellular pH measurements for planktonic mutant NCIB 3610 Δ*sinI*. Data: mean ± SD, *n* = 42 technical replicates. Strain: NCIB 3610 Δ*sinI*.** (g)** Comparison of microenvironment pH between 3610 WT and Δ*sinI* after 60 h of growth. Biofilm end pH values were statistically higher compared to planktonic mutant (*P* < 0.05). Data: *n* = 42 technical replicates per strain. Statistical significance was calculated using a Student’s *t*-test with *P* < 0.000001. Strains: NCIB 3610 and NCIB 3610 Δ*sinI*.

In the planktonic lifestyle, acidic metabolic byproducts produced via overflow metabolism can freely diffuse into the bulk medium, thereby minimizing local acidification (Fig. 1a) (32, 33). In contrast, in densely packed bacterial communities known as biofilms, acidic metabolic byproducts accumulate in the local environment due to limited diffusion (Fig. 1a) (34–36). In buffered laboratory media, such excessive acidification is counteracted by an external chemical buffer such as 3-(N-morpholino)propanesulfonic acid (MOPS; Fig. 1b, left) (25, 26). The external chemical buffer allows densely packed biofilms to continue proliferating despite the accumulation of acidic metabolic byproducts. While experimentally convenient, the common use of external chemical buffers in biofilm experiments provokes the question of how undomesticated biofilms in nature cope with largely minimally buffered conditions (Fig. 1b, right). Accordingly, we wondered whether biofilms have active strategies for mitigating the accumulation of acidic metabolic byproducts.

To approach this question, we established a minimally buffered experimental system capable of tracking extracellular pH during biofilm development. Specifically, we modified the defined media MSgg, commonly used to grow *B. subtilis* NCIB 3610 biofilms, by systematically varying each buffer component while monitoring growth and biofilm development. We found that reducing the MOPS buffer concentration from 100 to 1 mM while maintaining standard potassium-phosphate buffer levels permitted biofilm growth and development without measurable defect (Fig. 1c; Fig. S1a). To track biofilm pH, we utilized 10 μM 2′,7′-bis-(carboxyethyl)-5-(and-6)-carboxyfluorescein (BCECF) free acid, a cell-impermeable dye whose fluorescence linearly scales with the physiologically relevant pH range 5 to 9 (Fig. S2). We could then grow 3610 wild-type (WT) biofilms in static liquid MSgg (minimally buffered vs fully buffered) with 10 μM BCECF free acid at 30°C to form liquid-air pellicles and track BCECF fluorescence over 68 h. This experimental system permits the dynamic measurement of extracellular pH during biofilm development in a minimally buffered environment.

Using this experimental system, we found that *B. subtilis* displays a striking two-phase pH dynamic during biofilm development that is completely masked in standard MSgg media (Fig. 1d). Specifically, we observed an initial acidification phase (15.0 ± 0.3 h) followed by an extended alkalinization phase (31.2 ± 0.5 h) that ultimately returns the pH to the neutrophile range (Fig. 1e). Biofilms acidify to approximately pH 5.5 at an average rate of 0.06 ± 0.0008 pH/h and alkalinize back to pH 6.9 at an average rate of 0.03 ± 0.0005 pH/h (*n* = 42). We verified that this dynamic is not due to changes in growth rate (Fig. S1a) and observed that the alkalinization phase occurred during latter biofilm development. Furthermore, we found that a planktonic mutant strain (3610 ∆*sinI*) was unable to return to neutral (one-sided *t*-test, *n* = 42, *P* < 0.000001) due to a complete lack of the alkalinization phase (Fig. 1f and g). As before, we verified that the absence of alkalinization was not due to differences in growth (Fig. S1b). Thus, we concluded that the two-phase pH dynamic is specific to the biofilm lifestyle.

**Fig 2:**
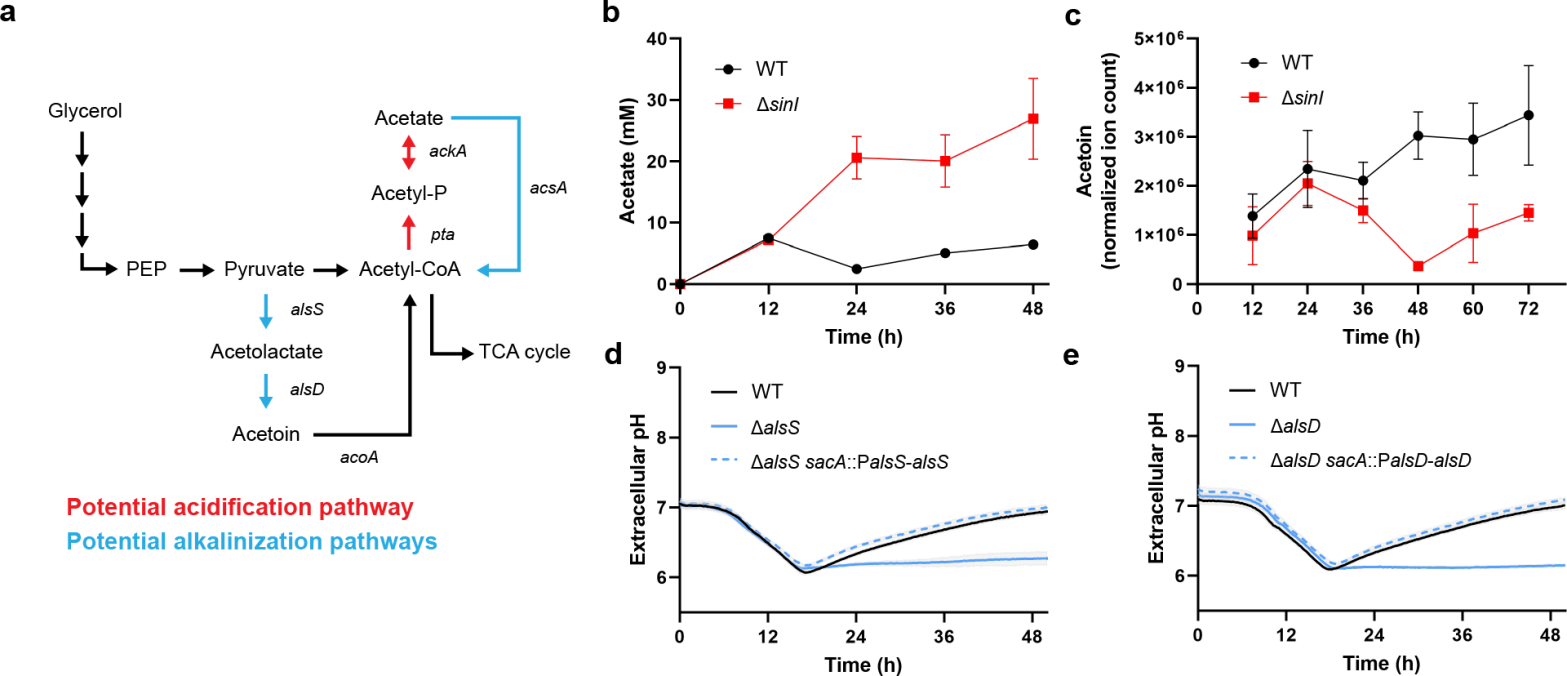
Genetic mechanisms responsible for extracellular pH modulation in *B. subtilis* biofilms. (**a)** Schematic showing acetate and acetoin metabolism in *B. subtilis* NCIB 3610 as potential sources for extracellular acidification and alkalinization. Potential acidification pathways are highlighted in red, whereas potential alkalinization pathways are highlighted in blue. (**b)** Acetate measurements of biofilm extracellular media. Data: mean ± SD, *n* = 3 technical replicates. Strains: NCIB 3610, Δ*sinI*.** (c)** Acetoin measurements of biofilm extracellular media as measured by metabolomic profiling. Data: mean ± SD, *n* = 3 technical replicates. Strains: NCIB 3610, Δ*sinI*. (**d)** Extracellular pH measurements for *alsS* mutant. Data: mean ± SD, *n* = 3 technical replicates. Strains: NCIB 3610, Δ*alsS*. (**e)** Extracellular pH measurements for *alsD* mutant. Data: mean ± SD, *n* = 3 technical replicates. Strains: NCIB 3610, Δ*alsD*.

We sought to determine the genes responsible for driving the observed acidification and alkalinization. We considered metabolic pathways and processes that could both acidify and alkalinize the biofilm environment (Fig. 2a; Fig. S3a). We first investigated ETC-associated complexes that can act as proton pumps and could drive acidification of the biofilm environment. We individually disrupted all five *B. subtilis* ETC complexes with known proton pump function and observed that no deletion produced significant change to the observed pH dynamic (Fig. S3b). In the case of ∆*ctaCD*, ∆*qoxA*, ∆*cydA*, and ∆*ythB* mutants, each deletion was a major subunit in the ETC complex resulting in total loss of function for that enzyme. These results suggest that common sources of direct proton transport are not entirely responsible for the observed pH dynamic. However, it is likely that multiple sources of metabolic acidification may contribute to the observed phenomenon.

We next suspected fermentation as a source of acidification since it is known that proliferating bacteria excrete acidic metabolites during overflow metabolism (17). We initially suspected lactate fermentation, as lactate is commonly produced during exponential and stationary growth to replenish redox carriers (37). However, a lactate production-deficient mutant (∆*ldh*) did not show any difference in acidification compared to WT (Fig. S3c). We then considered acetate fermentation, where acetate production similarly yields ATP and provides a substrate for the TCA cycle (Fig. 2a). To determine if acetate fermentation is involved in biofilm pH regulation, we first measured acetate levels in the extracellular environment during growth using a chemical acetate kit. We observed that biofilms transiently accumulate acetate before maturation, while planktonic mutants continue to accumulate larger quantities of acetate (Fig. 2b). We then generated mutants for each enzyme in the acetate biosynthesis pathway. While an *ackA* mutant (∆*ackA*) retained the acidification rate (Fig. S3d), a double mutant of *ackA* and *acsA* (∆*ack*A∆*acsA*), where *acsA* is an enzyme that can reversibly convert acetate into acetyl-CoA, had a reduced acidification rate. We quantified this reduction and observed that the ∆*ack*A∆*acsA* mutant had an acidification rate of approximately 48% less compared to that of wild type. Therefore, we concluded that acetate production is a significant source of acidification during biofilm development, while other sources of metabolic acidification remain.

On the other hand, to determine the genetic mechanism of alkalinization, we initially suspected ammonia as a critical community metabolite and known volatile alkaline species. However, deleting enzymes involved in ammonia synthesis produced no change to the alkalinization phase (Fig. S3e). We then considered acetoin biosynthesis as a pathway that has been speculated to circumvent lethal acidification via consumption of free protons (38–40). The acetoin pathway consists of two enzymatic conversion steps where AlsS (acetolactate synthase) converts pyruvate to acetolactate and AlsD (acetoin synthase) converts acetolactate to acetoin, with each step consuming a proton (Fig. 2a). Using metabolomics, we found that overall acetoin levels significantly increased in WT biofilms compared to those in the planktonic ∆*sinI* mutant (Fig. 2c). We then generated mutants for each step and observed that both the *alsS* (∆*alsS*) and *alsD* (∆*alsD*) mutants retained the acidification phase yet completely lost the alkalinization phase (Fig. 2d and e). Genetic complements of *alsS* and *alsD* in their respective mutant backgrounds showed restoration of the alkalinization phase. To explore whether acetoin catabolism would affect biofilm extracellular pH, we generated an *acoA* mutant and saw no difference in pH regulation compared to WT (Fig. S3f). Additionally, we found that acetoin itself (up to 50 mM) produces no change in the magnitude or timing of alkalinization in either WT or ∆*alsS*, suggesting that the process of acetoin biosynthesis itself, and not the acetoin product, was the main driver for biofilm alkalinization (Fig. S4a and b). Therefore, we concluded that the acetoin biosynthesis process is responsible for alkalinization.

**Fig 3:**
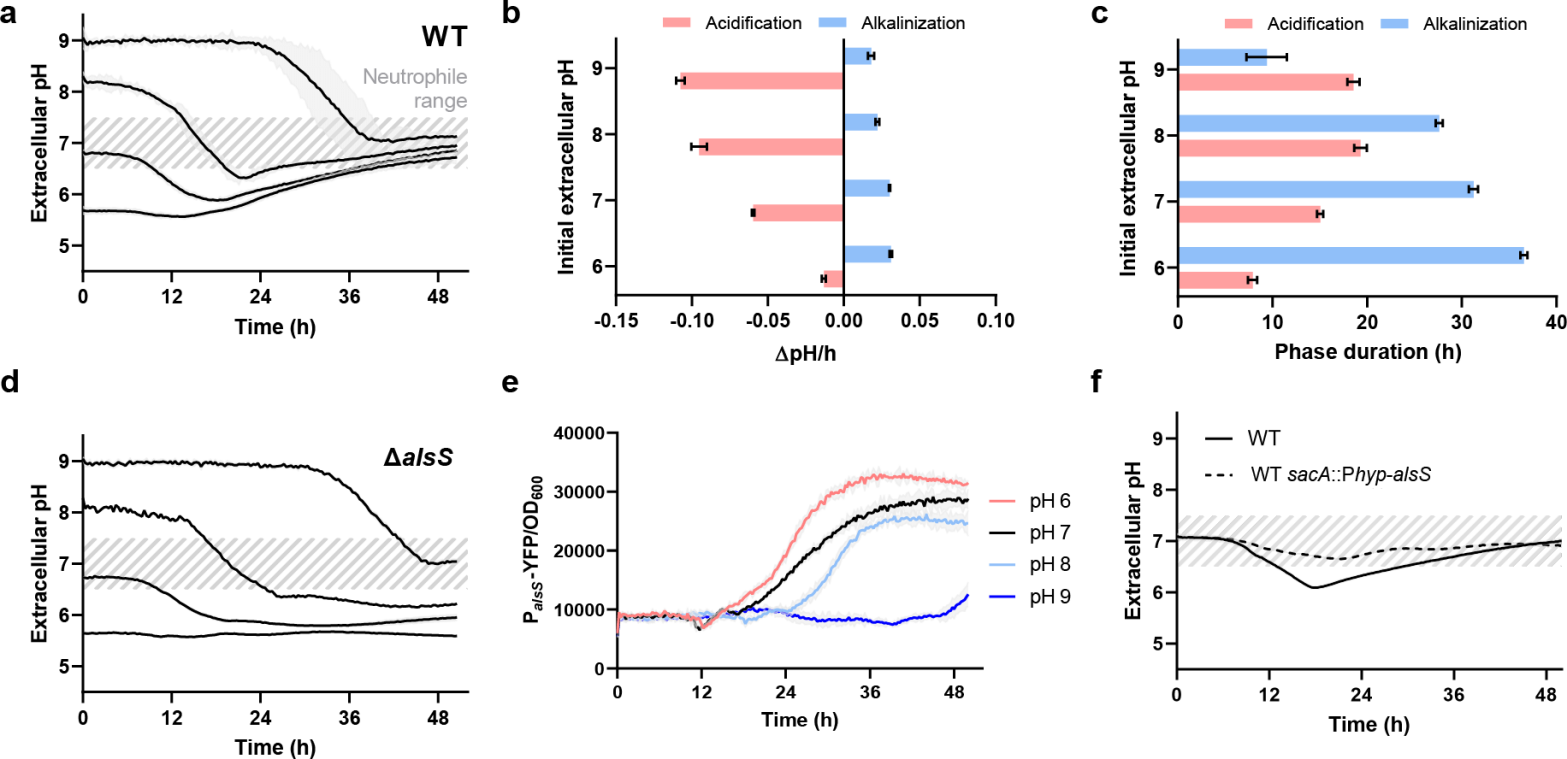
Active pH regulation during *B. subtilis* biofilm development via acetoin biosynthesis. (**a**) Measurement of extracellular pH for NCIB 3610 biofilms grown across a range of starting pH conditions (pH 6, 7, 8, 9). Dashed gray area represents optimal extracellular pH for neutrophile organisms. Biofilms were grown at 30°C statically. Data: mean ± SD, *n* = 3 technical replicates. (**b)** Average acidification and alkalinization rates for biofilm extracellular pH. Data from ΔpH/h traces were analyzed and averaged to determine biofilm acidification (<0 ΔpH/h) and alkalinization (>0 ΔpH/h). Data: mean ± SD, *n* = 3 technical replicates. (**c)** Phase duration for biofilm pH dynamics. Data from ΔpH/h traces were analyzed to determine biofilm acidification and alkalinization phase duration by measuring time periods where ΔpH/h was predominately <0 and >0, respectively. Data: mean ± SD, *n* = 3 technical replicates. (**d)** Measurement of extracellular pH for Δ*alsS* mutant biofilms grown across a range of starting pH conditions (pH 6, 7, 8, 9). Dashed gray area represents optimal extracellular pH for neutrophile organisms. Biofilms were grown at 30°C statically. Data: mean ± SD, *n* = 3 technical replicates. (**e)** Measurements of acetoin biosynthesis via genetically encoded reporters for acetolactate synthase (*alsS*). Data: mean ± SD, *n* = 3 technical replicates. Strain: NCIB 3610. (**f)** Measurement of extracellular pH for NCIB 3610 biofilms without and with inducible alsS expression grown starting at pH 7. Dashed gray area represents optimal extracellular pH for neutrophile organisms. Biofilms were grown at 30°C statically. Data: mean ± SD, *n* = 3 technical replicates. Strains: NCIB 3610, NCIB 3610 *sacA*::P*hyp-alsS*.

We then asked whether biofilms could utilize acetoin biosynthesis as an active pH regulation mechanism. We grew biofilms in minimally buffered MSgg media conditioned to a range of initial pH values (pH 6 to 9) and tracked the local pH and AlsS expression in each case (Fig. 3a). Strikingly, we found that biofilms conditioned their local pH to the preferred neutrophile range by modulating both the magnitude and duration of the alkalinization phase (Fig. 3b). Specifically, in acidic initial conditions (pH 6), alkalinization proceeded at a rate of 0.03 pH/h over a longer duration (36.6 ± 0.4 h) compared to neutral initial conditions (31.2 ± 0.5 h; Fig. 3c). Conversely, biofilms grown in basic conditions (pH 8 and 9) minimized alkalinization in both magnitude and duration (Fig. 3c). Interestingly, while the observed phases differed, biofilms grown at each pH condition produced similar matrix levels as indicated by safranin staining (Fig. S5). Comparatively, ∆*alsS* mutant biofilms failed to maintain their local pH in the preferred neutrophile range (Fig. 3d). In agreement with the pH measurements on WT biofilms, a P*_alsS_*-YFP reporter strain revealed that AlsS expression was increased in acidic conditions and decreased in alkaline conditions (Fig. 3e). Indeed, previous studies have shown that acetoin biosynthesis is upregulated during acidic conditions (39, 41–43). We then wondered if overexpression of the AlsS pathway could change the dynamics of the observed alkalinization phase. Using an inducible AlsS overexpression strain, we observed that AlsS overexpression decreases the time required to return to the neutrophile range (Fig. 3f). Our data reveals that biofilms can use acetoin biosynthesis as an active pH regulation mechanism to mitigate growth-associated acidification even in non-ideal pH environments.

**Fig 4:**
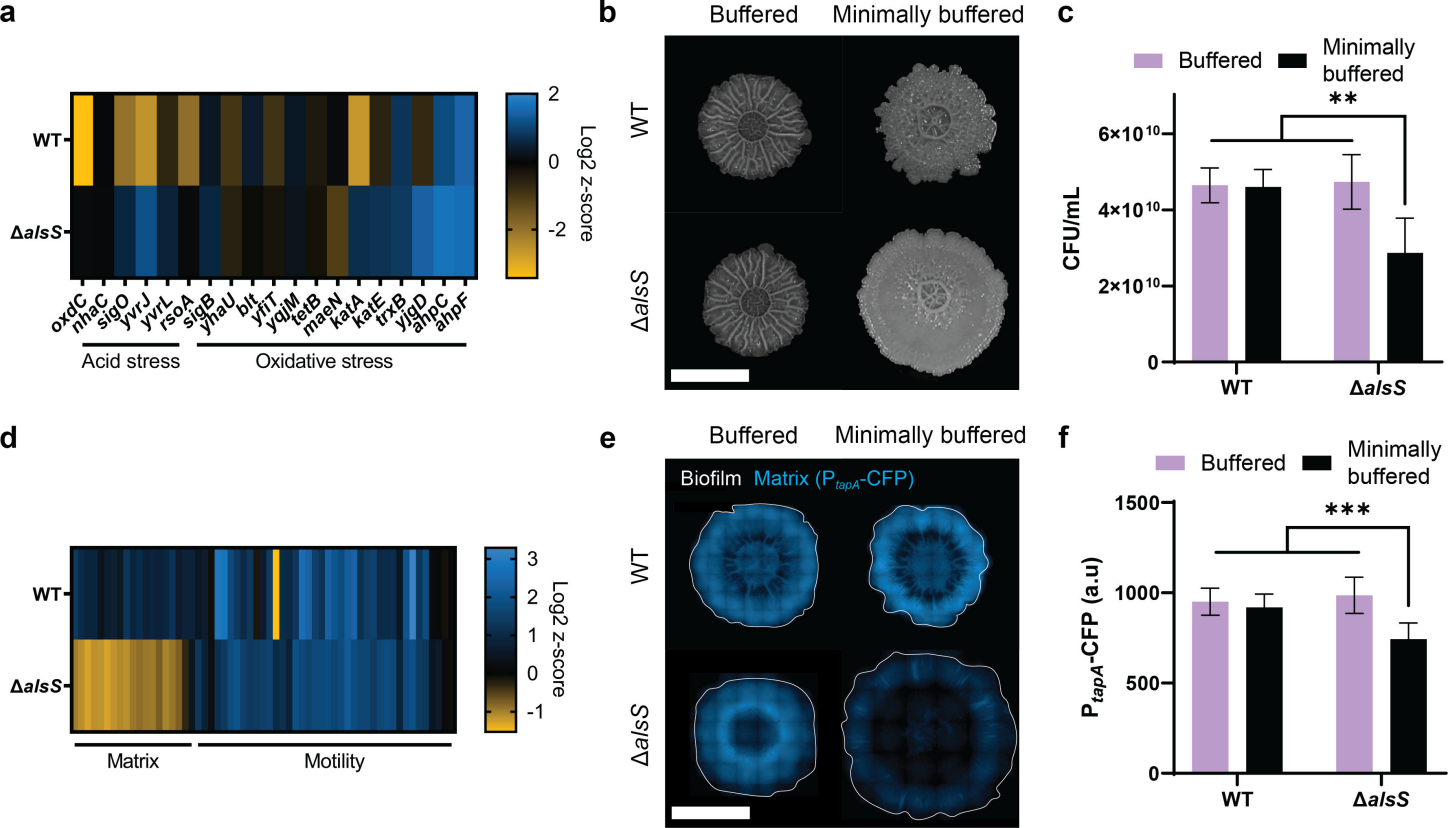
Physiological characterization of *B. subtilis* biofilms and buffering-deficient mutants (**a)** Heat map showing differentially expressed genes in acid and oxidative stress, induced by minimization of extracellular buffer, *n* = 3. (**b)** Images of *B. subtilis* NCIB 3610 WT and ∆*alsS* on buffered and minimally buffered MSgg solid agar, grown over 60 h. Scale bar represents 2 mm. (**c)** CFU measurements of 3610 WT and ∆*alsS* biofilms grown on buffered and minimally buffered MSgg solid agar harvested at 60 h. Data: mean ± SD, *n* = 6 technical replicates. (**d)** Heat map showing differentially expressed genes associated with matrix and motility in NCIB 3610 WT and ∆*alsS* biofilms induced by minimization of extracellular buffer, *n* = 3. (**e)** Fluorescence microscopy images of 3610 WT and ∆*alsS* biofilms expressing P*_tapA_*-CFP on buffered and minimally buffered MSgg solid agar at 36 h. White outline denotes biofilm edge, blue represents TapA matrix expression. Scale bar represents 2 mm. (**f)** Fluorescence measurements of 3610 WT and ∆*alsS* biofilms grown on buffered and minimally buffered MSgg solid agar harvested at 36 h. Data: mean ± SD, *n* = 6 technical replicates.

To validate acetoin biosynthesis as an active pH regulation mechanism, we performed RNA sequencing (RNAseq) on WT and ∆*alsS* mutant biofilms to identify differentially expressed genes (DEGs) in both normal and minimally buffered media. We confirmed that the *alsSD* pathway was upregulated in WT biofilms in minimally buffered media (Fig. S6). Interestingly, we found that ∆*alsS* mutant biofilms grown in minimally buffered media upregulated *ilvBH*, an alternate acetolactate synthase that could potentially compensate for loss of *alsS* activity (Fig. S6). As expected, we observed upregulation of several acid stress genes in ∆*alsS* mutant biofilms grown in minimally buffered media (Fig. 4a). In addition, we observed upregulation of several oxidative stress genes in ∆*alsS* mutant biofilms (Fig. 4a). This oxidative stress may result from a dysregulation of the PMF when active pH regulation is absent. Taken together, these results corroborate that biofilms utilize acetoin biosynthesis as a form of active pH regulation to maintain pH homeostasis and minimize cellular stress.

Accordingly, we wondered if active pH regulation could facilitate biofilm development in minimally buffered conditions. We first compared WT and ∆*alsS* biofilms on buffered media and found no significant difference in their overall growth and morphology (Fig. 4b, top). However, while WT biofilms largely maintained biofilm morphology in minimally buffered conditions, ∆*alsS* mutant biofilms lacked macroscopic wrinkles and displayed altered biofilm morphology, suggesting a difference in the biofilm ECM that maintains biofilm structure (Fig. 4b, bottom). In agreement with these observations, we found that ∆*alsS* biofilms had a significantly lower cell count (*P* < 0.001), which was specific to minimally buffered conditions, suggesting that inability to alkalinize via acetoin production was detrimental to biofilm growth (Fig. 4c). These results suggest that acetoin biosynthesis plays a role in biofilm development specific to minimally buffered environments.

To corroborate these findings, we performed RNAseq analysis to identify DEGs associated with biofilm development and ECM production. We found that ∆*alsS* mutant biofilms grown in minimally buffered media downregulated 16 of the 18 known ECM-associated genes in *B. subtilis,* while WT biofilms did not (Fig. 4d). We then created a genetically encoded fluorescent reporter for TapA, the anchoring and assembly protein for biofilm amyloid fiber. As expected, we found that WT biofilms displayed comparable TapA reporter expression in both buffered and minimally buffered conditions, in agreement with our prior observations on biofilm morphology (Fig. 4e, left). In contrast, while ∆*alsS* biofilms had comparable TapA reporter expression in buffered conditions, we measured significantly reduced (*P* < 0.0001) TapA expression in minimally buffered conditions (Fig. 4e, right, and f). Interestingly, both ∆*alsS* mutant biofilms and WT biofilms upregulated motility genes in minimally buffered conditions, suggesting the possibility of acidification-associated biofilm dispersal. Taken together, these results confirm that active pH regulation facilitates biofilm ECM formation in minimally buffered conditions.

## DISCUSSION

**Fig 5:**
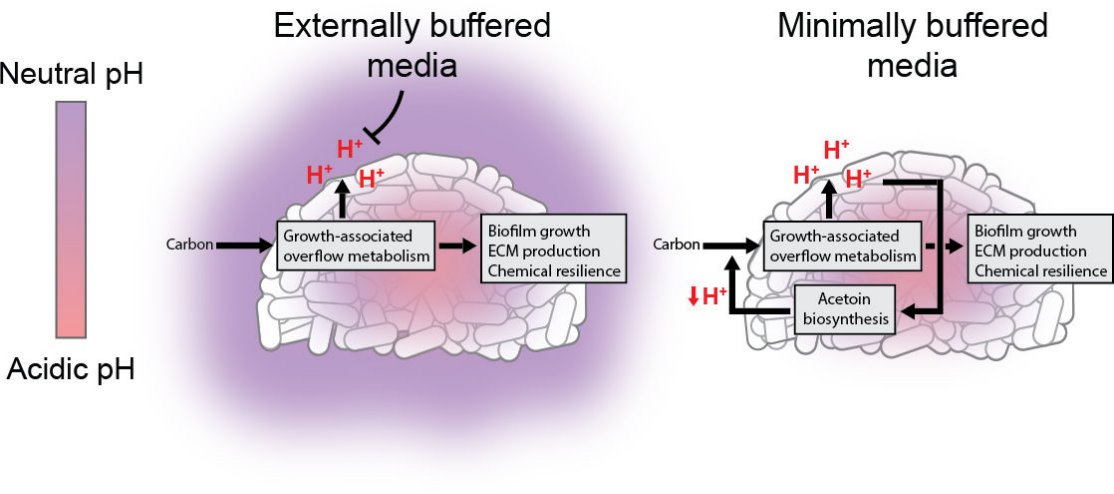
Proposed schematic of active pH regulation in *B. subtilis* biofilms.

While chemical buffers can be employed to study biofilms in the laboratory, they can also mask the underlying biology of pH management during biofilm development. In our study, we established a minimally buffered system to determine how undomesticated *B. subtilis* biofilms cope with growth-associated acidification. We discovered an active pH regulation mechanism that effectively mitigates both growth-associated acidification and external pH challenges. This phenomenon is fully masked in buffered laboratory media—such as MSgg commonly used to study *Bacillus subtilis* biofilms—and relies on the pH-dependent expression of acetoin biosynthesis. Disruption of acetoin biosynthesis results in dysregulated biofilm development and decreased extracellular matrix production. Thus, this active pH regulation mechanism enables biofilms to minimize cellular stresses and maintain community resilience against both internal growth-associated acidification and external pH challenges (Fig. 5). The discovery of active pH regulation in *B. subtilis* biofilms could provide new opportunities for understanding microbial communities, controlling pathogenic biofilm growth, and engineering novel biofilm behaviors.

Additional studies will be needed to determine whether this active pH regulation mechanism is found in other biofilm-forming species. While acetoin biosynthesis is largely conserved across both Gram-positive and Gram-negative bacteria, the pH-dependent expression of these pathways, especially in minimally buffered conditions, remains an open question. Furthermore, while overflow metabolism has been well characterized in planktonic lab strains of *Escherichia coli*, its potential impact on biofilm development and pH regulation remains unclear (17, 44). We propose that overflow metabolism, specifically acetoin biosynthesis, provides a critical detoxification pathway for immobilized and densely packed communities during biofilm development. Our proposal aligns with and extends mechanistic single-cell level studies that reveal the potential role of acetoin biosynthesis in mitigating local acidification (45).

Future studies may also elucidate how active pH regulation influences other emergent behaviors observed in biofilms. For example, in natural environments, such as soil, geothermal springs, and human gastrointestinal tract, variations and gradients in pH give rise to unique bacterial behaviors such as extracellular electron transport, chemotropy, and increased drug resistance (46–48). Our results are also intriguing to consider alongside recent results that show acetate biosynthesis pathway promoting biofilm development over macroscopic length scales (49, 50). As biofilm growth appears to necessitate acetate production, it would be interesting to determine how local acetate production influences nearby communities and how each community integrates their local needs with those of their neighbors. Active pH regulation could also help stabilize the PMF of biofilm cells during electrochemical signaling, enabling cells to modulate ionic efflux and membrane voltage while maintaining cellular growth.

## MATERIALS AND METHODS

### Growth conditions

Bacteria were grown in Luria–Bertani (LB) rich media overnight and/or grown on the day of experiment and seeded into MSgg media. Biofilms in fully buffered conditions were grown in standard MSgg, which contains 100 mM MOPS, 5 mM potassium-phosphate buffer (pH 7), 2 mM MgCl_2_, 700 µM CaCl_2_, 50 µM MnCl_2_, 100 µM FeCl_3_, 1 µM ZnCl_2_, 2 µM thiamine HCl, 0.5% (vol/vol) glycerol, and 0.5% (wt/vol) monosodium glutamate. Biofilms in minimally buffered conditions were grown in modified MSgg, which contains 1 mM MOPS, 5 mM potassium phosphate buffer (pH 7), 2 mM MgCl_2_, 700 µM CaCl_2_, 50 µM MnCl_2_, 100 µM FeCl_3_, 1 µM ZnCl_2_, 2 µM thiamine HCl, 0.5% (vol/vol) glycerol, and 0.5% (wt/vol) monosodium glutamate. To measure biofilm extracellular pH, 10 µM BCECF free acid (Biotium) was used in MSgg. Strains were grown to optical density 0.8–1.0 in LB, spun down and resuspended in 1× PBS. One microliter of cell culture was then seeded into 199 µL of MSgg in a 96-well microplate (Corning 3904) or into a well with 0.6 mL of solid MSgg agar in a 24-well plate (Corning 3526).

### Optical density, fluorescence, and cell density measurements

Optical density (530 and 600 nm), BCECF, and YFP fluorescence in all our studies were measured using a TECAN Infinite MPLEX plate reader with excitation/emission wavelength set to 503/530 nm and gain set to 100. To quantify biofilm extracellular pH, minimally buffered MSgg was prepared for each experiment and conditioned to pH 5, 6, 7, 8, and 9 using 1 M HCl and 1 M NaOH when appropriate. BCECF dye was then added to these aliquots and included as a standard curve to convert the measured BCECF fluorescence signal to extracellular pH. Cell density of biofilms or planktonic cultures was quantified using a hemocytometer or Logos Biosystems Quantom TX Microbial Counter. To prepare biofilms for cell density quantification, biofilms were grown for the desired time in either buffered or minimally buffered MSgg media. Each biofilm was harvested into 1 mL of 1× PBS solution and sonicated for 5 s on ice using a Qsonica Q125 125W 20-kHz sonicator at 60% amplitude. For hemocytometer counting, the resulting cell suspension was fixed with paraformaldehyde, diluted into PBS, and counted using phase microscopy. For the Quantom TX, the cell suspension was diluted in PBS, stained using the Logos Total Cell Staining kit, and imaged directly on the Quantom TX.

### DNA cloning

Custom promoter sequences were ordered from Integrated DNA Technologies (IDT) or amplified from the native NCIB genome and cloned upstream of a YFP reporter in a *B. subtilis* integration vector ECE174 (https://bgsc.org/search.php?Search=ece174) with chloramphenicol resistance. All plasmid assembly was performed using Gibson Assembly using the Gibson Assembly Master Mix (NEB). The assembled plasmid was transformed into NCIB 3610 using a natural competence protocol previously described and plated on LB agar with appropriate selection (51). Genetic complements with native promoters were amplified from the native NCIB genome with 500 bp of the native promoters and added to the integration vector ECE174 with chloramphenicol resistance. Gene overexpression was amplified the same way as genetic complements, but the native promoter was swapped with pHyperspank for inducible expression of the given gene with isopropyl β-D-1-thiogalactopyranoside. Gene overexpression constructs were also added to ECE174 with chloramphenicol resistance.

### Acetate measurements

Biofilms (3,610) were grown in minimally buffered MSgg media in a 96-well microplate (Corning 3904). Supernatant was harvested at given timepoints and stored at −80°C until all samples were collected. Samples were transferred to microcentrifuge tubes and cleared of debris by centrifugation at 20,000*g* for 15 min at 4°C. Sample supernatants were transferred to fresh tubes and diluted with water to be within the working range of the kit. Acetate was measured using the Abnova KA3780 kit according to protocol provided. Measurements were conducted in the colorimetric range.

### Biofilm growth and normalization for metabolomics

Bacteria were grown in LB rich media on the day of experiment, washed with PBS, and seeded onto MSgg media agar pads in six-well plates. Biofilms were harvested at given timepoints into 1 mL of −80°C prechilled 80% methanol. Samples were then frozen at −80°C until all samples were collected. Samples were then sonicated on ice for metabolite extraction using a Qsonica Q125 125W 20 kHz sonicator. Sonication was performed at 60% amplitude for 45 s—one 30-s sonication followed by a 15-s sonication with at least 5 min of rest on ice in between. Protein from lysed samples was precipitated at −80°C overnight. Samples were then cleared of debris by centrifugation at 20,000*g* for 15 min at 4°C. Sample supernatants were transferred to Northwestern’s Core Facility for Samples Reconstitution and Metabolomic analysis. For normalization, replicate biofilms were grown and harvested for every timepoint to determine total biofilm cell counts (11). Biofilms were collected in ice-cold PBS, sonicated for 5 s to disrupt cells from the matrix, and diluted 20× in PBS. Biofilm cell counts were then determined using Logos Total Cell Count staining kit and imaged directly on Quantom TX. Cell counts were used for normalization in “Metabolomics sample reconstitution after extraction” and “Metabolomics” below.

### Metabolomics sample reconstitution after extraction

Extraction solution was dried using SpeedVac. Acetonitrile (50%) was added to the tube for reconstitution followed by overtaxing for 30 s. Sample solution was then centrifuged for 30 min at 20,000*g*, 4°C. Supernatant was collected for liquid chromatography-mass spectrometry analysis.

### Metabolomics

Samples were analyzed by high-performance liquid chromatography and high-resolution mass spectrometry and tandem mass spectrometry (HPLC-MS/MS) (52, 53). Specifically, the system consisted of a Thermo Q-Exactive in line with an electrospray source and an Ultimate3000 (Thermo) series HPLC consisting of a binary pump, degasser, and auto-sampler outfitted with an Xbridge Amide column (Waters; dimensions of 3.0 mm × 100 mm and a 3.5-µm particle size). The mobile phase A contained 95% (vol/vol) water, 5% (vol/vol) acetonitrile, 10 mM ammonium hydroxide, 10 mM ammonium acetate, pH = 9.0; B was 100% acetonitrile. The gradient was as follows: 0 min, 15% A; 2.5 min, 30% A; 7 min, 43% A; 16 min, 62% A; 16.1–18 min, 75% A; 18–25 min, 15% A with a flow rate of 150 µL/min. The capillary of the ESI source was set to 275°C, with sheath gas at 35 arbitrary units, auxiliary gas at 5 arbitrary units, and the spray voltage at 4.0 kV. In positive/negative polarity switching mode, an *m*/*z* scan range from 60 to 900 was chosen, and MS1 data were collected at a resolution of 70,000. The automatic gain control (AGC) target was set at 1 × 10^6^, and the maximum injection time was 200 ms. The top five precursor ions were subsequently fragmented, in a data-dependent manner, using the higher-energy collisional dissociation (HCD) cell set to 30% normalized collision energy in MS2 at a resolution power of 17,500. Besides matching *m*/*z*, metabolites are identified by matching either retention time with analytical standards and/or MS2 fragmentation pattern. Data acquisition and analysis were carried out by Xcalibur 4.1 software and Tracefinder 4.1 software, respectively (both from Thermo Fisher Scientific).

### RNA isolation

3610 biofilms were grown for 36 h in either buffered or minimally buffered MSgg media. Each biofilm was harvested into 1 mL of pre-chilled 50% methanol solution. The biofilm was then pelleted by centrifugation, aspirated to remove supernatant, and flash frozen with liquid nitrogen before being stored overnight at -80°C. RNA was then isolated using the QIAGEN RNeasy kit (QIAGEN) according to the manufacturer’s instructions with lysis being completed by 30s of bead-beating using Lysis Matrix B tubes in the Omni Bead Ruptor Elite bead beating machine.

### RNA sequencing

RNA quality was checked using Bioanalyzer (Agilent) prior to RNA-seq library preparation. RNA samples with an RNA integrity number >8 were used for library preparation, which was constructed from 100 ng of RNA with the Illumina Stranded Total RNA Prep, Ligation with Ribo-Zero Plus kit (Illumina). RNA sequencing was then performed on a NovaSeq 6000 sequencer and analyzed as previously described. The quality of reads, in FASTQ format, was evaluated using FastQC. Reads were trimmed to remove Illumina adapters from the 3′ ends using cutadapt (54). Trimmed reads were aligned to the *B. subtilis* genome strain 3610 National Center for Biotechnology Information (NCBI) CP020102.1 and plasmid NCBI CP020103.1 using STAR (55). Read counts for each gene were calculated using htseq-count (Anders et al., 2015) in conjunction with a gene annotation file for the reference genomes obtained from NCBI. Normalization and differential expression were calculated using DESeq2, which employs the Wald test (56). The cutoff for determining significantly differentially expressed genes was an FDR-adjusted *P*-value < 0.05 using the Benjamini–Hochberg method.

### Microscopy

Biofilm growth was recorded using phase-contrast and fluorescence microscopy. The microscope used was a Nikon Ti2. To image entire biofilms, we used a 10× objective and the stitching function in Nikon Elements to assemble images. Images were taken every hour. Whenever fluorescence images were recorded, we used the minimum exposure time that still provided a good signal-to-noise ratio.

### Image analysis

Fiji/ImageJ (National Institutes of Health) was used for image analysis. To measure biofilm fluorescence, we identified the biofilm area first using phase and creating custom regions of interests (ROIs) that outlined the biofilm for each frame. We then used the same ROIs on the relevant fluorescent channel of the same experimental run to measure average fluorescent reporter signal over time.

### Statistical analyses

Statistical tests were calculated in GraphPad Prism 9.0. For comparisons between two independent groups, a Student’s *t*-test was used. Significance was accepted at *P* < 0.05. The details of the statistical tests carried out are indicated in respective figure legends.

## Data Availability

Data are available on NCBI GEO with accession number GSE231939.

## SUPPLEMENTAL MATERIAL

Supplemental Figures and Tables mbio.03387-23-s0001.docx Figures S1 to S6 and Tables S1 and S2.

ASM does not own the copyrights to Supplemental Material that may be linked to, or accessed through, an article. The authors have granted ASM a non-exclusive, world-wide license to publish the Supplemental Material files. Please contact the corresponding author directly for reuse.
