# Supplementary material for: Active pH regulation facilitates Bacillus subtilis biofilm development in a minimally buffered environment
Source: mBio. 2024 Feb 13;15(3):e03387-23. doi: 10.1128/mbio.03387-23 (PMC10936434; doi:10.1128/mbio.03387-23)
Supplement: Supplemental Figures and Tables — Figures S1 to S6 and Tables S1 and S2. [file mbio.03387-23-s0001.docx]

**Supplementary materials**


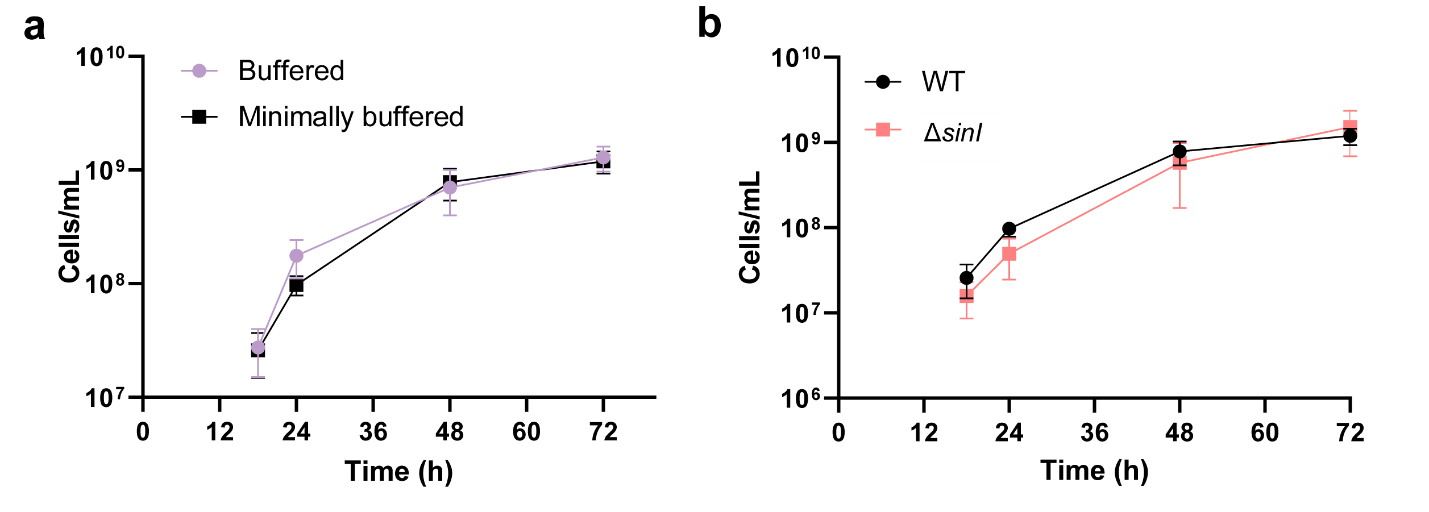


**Supplemental Figure 1: Biofilm growth measurements in buffered (100 mM MOPS) and minimally buffered (1 mM) MSgg media over time**. **a,** Biofilm cell density measurements over time. Biofilms were harvested in protocol outlined in Methods section. Data: mean ± std, n=3 technical replicates. **b,** 3610 WT and ∆*sinI* cell density measurements in minimally buffered (1 mM MOPS) MSgg. Both strains were harvested in protocol outlined in Methods section. Data: mean ± std, n=3 technical replicates.

**Supplemental Figure 2: BCECF free acid fluorescence over extracellular pH.** BCECF free acid was added into MSgg media at 10 uM concentration and measured at 490/535 nm Ex/Em, Red data points represent BCECF measurements taken at 0 h (directly after media preparation) and blue data points represent BCECF measurements taken after approximately 65.9 h in MSgg media incubated at 30°C. Both data sets were fitted with a simple linear regression with R^2^ = 0.94 in both cases. For each experiment, a BCECF standard curve was used to internally calibrate BCECF signal to pH and subsequently convert measured BCECF fluorescence during biofilm growth to extracellular pH.


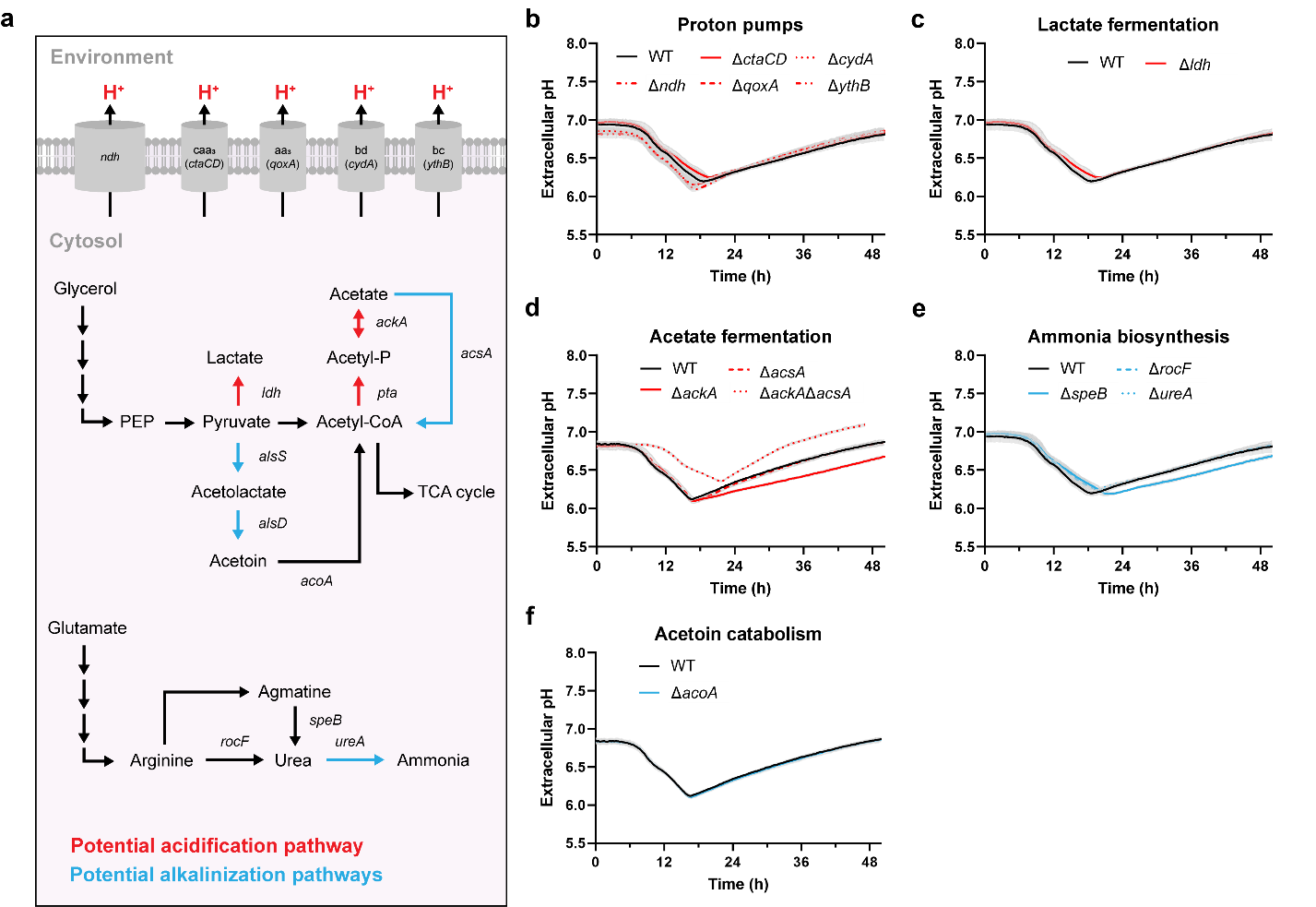


**Supplemental Figure 3: Genetic mechanisms responsible for extracellular pH modulation in biofilms.**

**a**, Schematic showing metabolic pathways in *B. subtilis* NCIB 3610 that are potential sources for extracellular acidification and alkalinization. Potential acidification pathways are highlighted in red whereas potential alkalinization pathways are highlighted in blue. **b**, Extracellular pH measurements for proton pump mutants. Data: mean ± std, n=3 technical replicates. Strains: NCIB 3610, Δ*ctaCD*, Δ*cydA*, Δ*ndh*, Δ*qoxA*, Δ*ythB*. **c**, Extracellular pH measurements for lactate dehydrogenase mutant, ∆*ldh*. Data: mean ± std, n=3 technical replicates. Strains: NCIB 3610, Δ*ldh*. **d**, Extracellular pH measurements for acetate biosynthesis and catabolism mutants. Data: mean ± std, n=3 technical replicates. Strains: NCIB 3610, Δ*ackA*, Δ*ackA*Δ*acsA*, Δ*acsA*, **e**, Extracellular pH measurements for ammonia biosynthesis mutants. Data: mean ± std, n=3 technical replicates. Strains: NCIB 3610, Δ*rocF*, Δ*speB*, Δ*ureA*. **f**, Extracellular pH measurements for acetoin catabolism mutant. Data: mean ± std, n=3 technical replicates. Strains: NCIB 3610, Δ*acoA.*


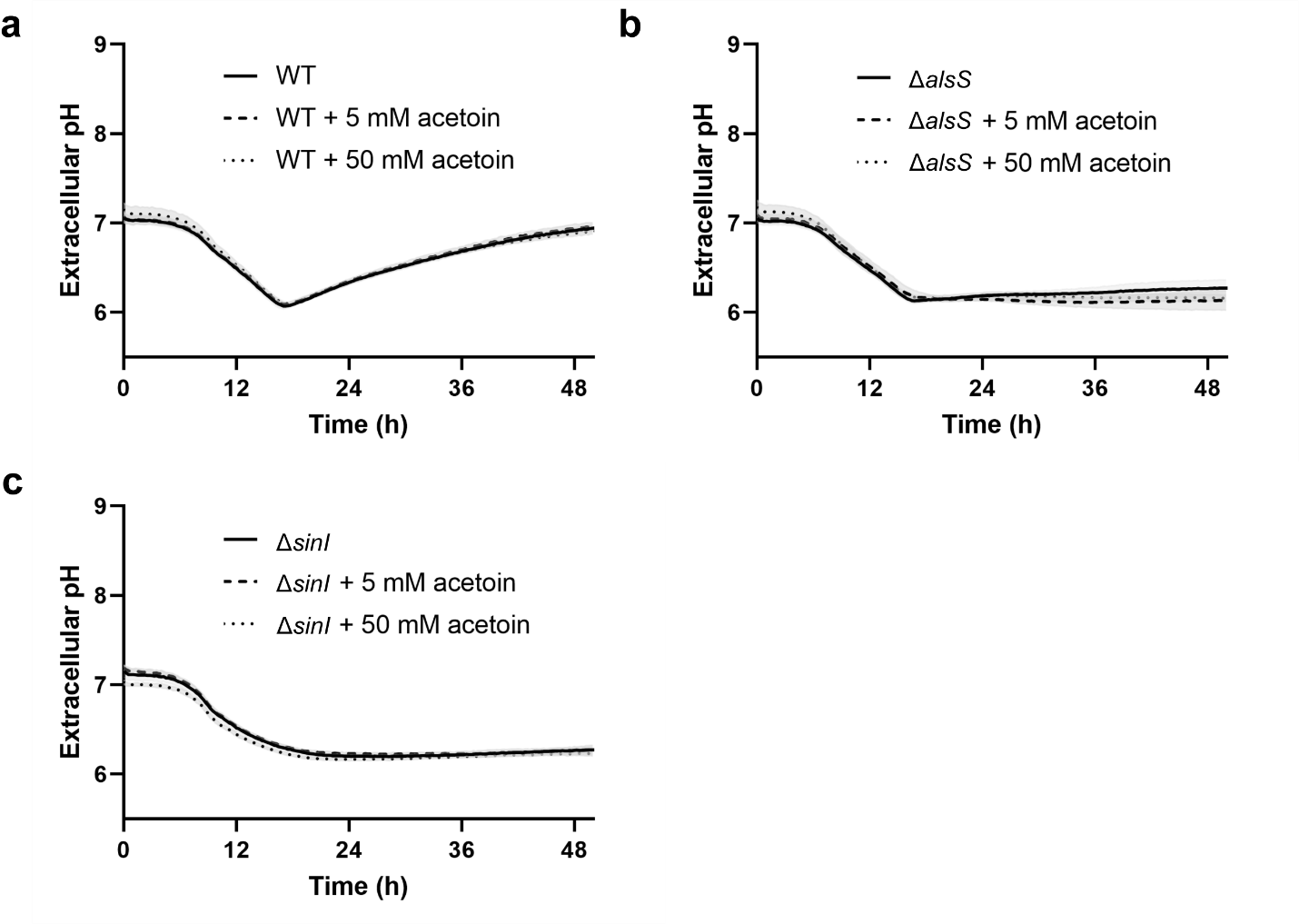


**Supplemental Figure 4: Extracellular pH measurements for biofilms grown in the presence of exogenous acetoin.** **a**, Acetoin added to the starting minimally buffered MSgg with 10 uM BCECF at 0, 5, and 50 mM concentrations and inoculated with NCIB 3610 at 30°C. Biofilms were grown for approximately 50 h. Data: mean ± std, n=3 technical replicates. **b**, Acetoin added to the starting minimally buffered MSgg with 10 uM BCECF at 0, 5, and 50 mM concentrations and inoculated with NCIB 3610 Δ*alsS* at 30°C. Biofilms were grown for approximately 50 h. Data: mean ± std, n=3 technical replicates. **c**, Acetoin added to the starting minimally buffered MSgg with 10 uM BCECF at 0, 5, and 50 mM concentrations and inoculated with NCIB 3610 Δ*sinI* at 30°C. Strain was grown for approximately 50 h. Data: mean ± std, n=3 technical replicates.

**Supplemental Figure 5: Safranin staining of biofilms grown at various pH starting conditions in minimally buffered MSgg.** Biofilms were allowed to grow for approximately 68 h at 30°C. Biofilm matrix was isolated via aspiration of remaining media and air dried overnight at room temperature. Matrix was then stained with safranin solution for 10 min, washed three times with water, and allowed to dry for another 30 minutes. 30% acetic acid was added to the matrix and allowed to stain for 30 minutes before measuring final optical density of the stained matrix at 530 nm.


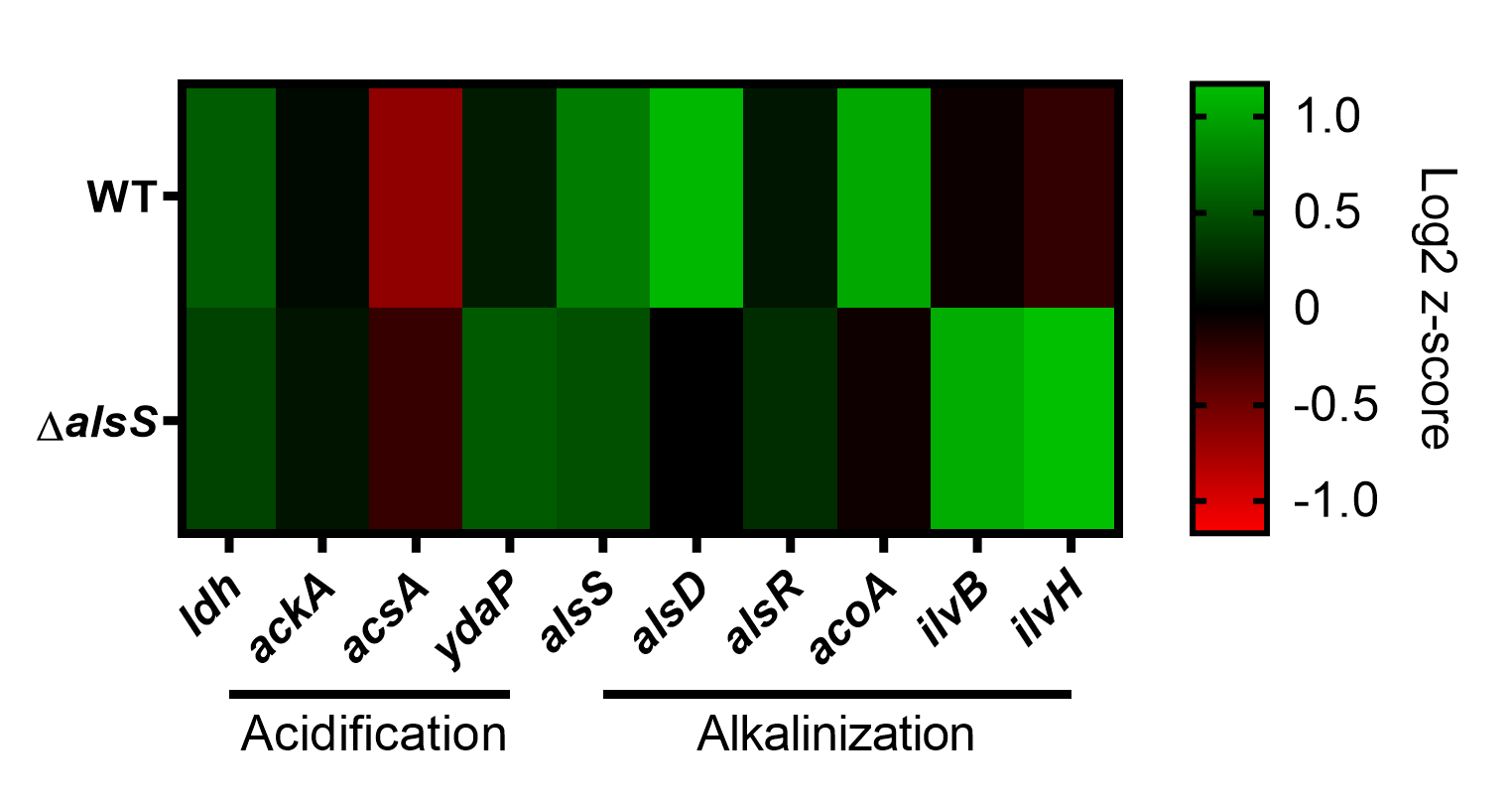


**Supplemental Figure 6: Transcriptomic analysis of acidification- and alkalinization-associated genes.** Heat map showing differentially expressed genes in overflow metabolism and potential candidates for extracellular acidification and alkalinization, induced by minimization of extracellular buffer, n=3

**Supplemental Table 1: Strains used in this study.**

| **Strain** | Organism | Genotype |
| --- | --- | --- |
| Wild type | *B. subtilis* NCIB 3610 |  |
| Δ*sinI* | *B. subtilis* NCIB 3610 | *sinI::neo* |
| Δ*ndh* | *B. subtilis* NCIB 3610 | *ndh::kan* |
| Δ*ctaCD* | *B. subtilis* NCIB 3610 | *ctaCD::neo* |
| Δ*cydA* | *B. subtilis* NCIB 3610 | *cydA::neo* |
| Δ*qoxA* | *B. subtilis* NCIB 3610 | *qoxA::neo* |
| Δ*ythB* | *B. subtilis* NCIB 3610 | *ythB::neo* |
| Δ*ldh* | *B. subtilis* NCIB 3610 | *ldh::kan* |
| Δ*ackA* | *B. subtilis* NCIB 3610 | *ackA::kan* |
| Δa*csA* | *B. subtilis* NCIB 3610 | *acsA::kan* |
| Δ*ackA* Δ*acsA* | *B. subtilis* NCIB 3610 | *ackA::acsA::kan* |
| Δ*rocF* | *B. subtilis* NCIB 3610 | *rocF::kan* |
| Δ*speB* | *B. subtilis* NCIB 3610 | *rocF::kan* |
| Δ*ureA* | *B. subtilis* NCIB 3610 | *rocF::kan* |
| Δ*alsS* | *B. subtilis* NCIB 3610 | *alsS*::*kan* |
| Δ*alsS sacA::*P*_alsS_-alsS* | *B. subtilis* NCIB 3610 | *sacA*:: P*_alsS_-alsS* |
| Δ*alsD* | *B. subtilis* NCIB 3610 | *alsD*::kan |
| Δ*alsD sacA*::P*_alsD_-alsD* | *B. subtilis* NCIB 3610 | *sacA*:: P*_alsD_*-alsD |
| Δ*acoA* | *B. subtilis* NCIB 3610 | *acoA:*:kan |
| P*_alsS_*-YFP | *B. subtilis* NCIB 3610 | *sacA*::P*_alsS_*-YFP |
| P*_hyp_*-alsS | *B. subtilis* NCIB 3610 | *sacA*::P*_hyp_*-*alsS* |
| P*_hag_*-mCherry, P*_tapA_*-CFP | *B. subtilis* NCIB 3610 | *amyE*:: P*_hag_*-mCherry, *sacA*::P*_alsS_*-YFP |
| Δ*alsS* P*_hag_*-mCherry, P*_tapA_*-CFP | *B. subtilis* NCIB 3610 | *alsS*::*kan , amyE*:: P*_hag_*-mCherry, *sacA*::P*_alsS_*-YFP |

**Supplemental Table 2: Planktonic doubling times for NCIB 3610 and acetoin mutants.**

|  | **LB t_d_ (min)** | | | |
| --- | --- | --- | --- | --- |
| **Strain** | **pH 6** | **pH 7** | **pH 8** | **pH 9** |
| WT | 119.2 ± 4.7 | 118.7 ± 10.6 | 122.8 ± 10.0 | 125.1 ± 10.7 |
| Δ*sinI* | 118.0 ± 8.7 | 117.5 ± 3.4 | 119.2 ± 1.6 | 121.9 ± 10.12 |
| Δ*alsS* | 122.4 ± 15.8 | 121.6 ± 8.7 | 121.4 ± 10.2 | 124.7 ± 2.7 |
| Δ*alsD* | 118.0 ± 6.7 | 123.0 ± 1.7 | 125.4 ± 10.6 | 124.2 ± 3.6 |
